# Glucose-lowering potential of *Guiera senegalensis* roots in a diabetic rat model

**Published:** 2020

**Authors:** David Miaffo, Fidèle Ntchapda, Oulianovie Guessom Kamgue, Abba Talba Mahamad, Albert Kamanyi

**Affiliations:** 1 *Laboratory of Physiology, Department of Life and Earth Sciences, Higher Teachers’ Training College, University of Maroua. * *P.O. Box 55 Maroua, Cameroun*; 2 *Laboratory of Biology, Department of Biological Sciences, Faculty of Science, University of Ngaoundéré, Cameroon. P.O. Box 454 Ngaoundéré, Cameroon*; 3 *Laboratory of Animal Physiology and Phytopharmacology, Department of Animal Biology, Faculty of Sciences, University of Dschang. P.O. Box 67 Dschang, Cameroon*; 4 *Laboratory of Biology, Department of Biological Sciences, Faculty of Sciences, University of Maroua, Cameroon. P.O. Box 814 Maroua, Cameroun*

**Keywords:** Guiera senegalensis, Lipid profile, Glycaemia, Diabetes mellitus, Phytochemical analysis

## Abstract

**Objective:**

*Guiera senegalensis* is distributed in the Sudano-Sahelian zone and used traditionally for the treatment of diabetes. This study was designed to assess the hypoglycemic effects of *G. senegalensis* in Wistar diabetic rats.

**Materials and Methods:**

Phytochemical analysis was carried out on aqueous and methanolic extracts of *G. senegalensis*. Type 2 diabetes was induced in male rats using nicotinamide/streptozotocin (65 mg/kg/110 mg/kg, i.p.). After diabetes induction, normal and negative control groups received distilled water, positive control group received glibenclamide (0.25 mg/kg) and the others group received aqueous and methanolic extracts (200 and 400 mg/kg, each) orally for 4 weeks. Glycaemia, body weight, insulin level, total cholesterol (TC), high density lipoprotein cholesterol (HDL-c), low density lipoprotein cholesterol (LDL-c), triglycerides (TG), aspartate amino transferase (AST) and alanine amino transferase (ALT) activities, urea and creatinine (Cr) were evaluated.

**Results:**

The content of phenols, flavonoids and tannins were 34.54 mg gallic acid equivalent (GAE)/gE, 4.86 mg quercetin equivalent (QE)/gE and 16.81 mg catechin equivalent (EC)/gE in the aqueous extract, respectively. Phenol (26.01 mg GAE/gE), flavonoid (4.47 mg QE/gE) and tannin (7.67 mg EC/gE) contents were also obtained for the methanolic extract. *G. senegalensis* and glibenclamide resulted in a significant increase (p<0.001) in body weight and HDL-c in diabetic group rats receiving glibenclamide and different doses of extracts. . The level of insulin, glycaemia, TG, TC, LDL-c, urea and creatinine significantly decreased (p<0.05 to 0.001) in diabetic animals treated with *G. senegalensis* extracts.

**Conclusion:**

These results confirm the potential of *G. senegalensis* for the treatment of diabetes and its complications.

## Introduction

Type 2 diabetes mellitus (T2DM) is a state of chronic hyperglycemia characterized by insulin deficiency associated with insulin resistance (Rang et al., 2007 ▶). It generally appears from the age of 40 and represents 85 to 90% of diabetics. T2DM is a serious disease that, without appropriate treatment, may be the cause of cardiovascular diseases (Gandhi et al., 2014 ▶). This carbohydrate metabolism disorder is a real global public health problem and affects all social strata regardless of sex or age. Indeed, in the world, diabetes-related deaths were about 1.5 million in 2012 and will reach about 3.7 million in 2040 (WHO, 2016 ▶).

Current diabetes treatments rely primarily on daily insulin injections and/or oral antidiabetic drugs. These antidiabetics have many adverse side effects and significant risks of cardiovascular diseases. In addition, some of them are contraindicated in pregnancy and in diabetic patients suffering from liver, cardiac and/or renal diseases (Surya et al., 2014 ▶). These limits push most of the world's population towards herbal medicine as an alternative for effective treatment, with low-toxicity and few adverse side effects. 

*Guiera senegalensis* (Combretaceae) is a plant that grows up to 3 m high and is present in the Sudano-Sahelian zone; it is found in Sudan, Senegal, Chad and Cameroon (Sombié et al., 2011 ▶). The leaves and roots of this plant are traditionally used for the treatment of diabetes and high blood pressure (Fiot et al., 2006 ▶). Previous studies showed the hypoglycemic potential of the ethanolic extract of the leaves of *G. senegalensis* in normal rats (Chahinez et al., 2012 ▶). The studies carried out by Osibemhe et al. (2018) ▶ and Sombié et al. (2019) ▶ showed the antidiabetic effect of *G. senegalensis* leaves in a streptozotocin-induced diabetes model. Yakubu et al. (2018) ▶ showed that administration of ethanolic leaf extract of *G. senegalensis* to alloxanized diabetic rats led to hypoglycemic activities. In addition, Miaffo et al. (2019) ▶ showed that the aqueous extract of *G. senegalensis* roots has the ability to reduce postprandial glucose and prevent insulin resistance in mice. However, no previous antidiabetic study was performed with the roots of *G. senegalensis* in the streptozotocin/nicotinamide-induced diabetes model. Thus, this study assessed the hypoglycemic potential of *G. senegalensis* roots in nicotinamide/streptozotocin-induced diabetic rats.

## Materials and Methods

**Chemicals and reagents**

Glibenclamide, streptozotocin, nicotinamide, Folin-Ciocalteu reagent, sodium carbonate, sodium nitrite, aluminium chloride, sodium hydroxide, hydrogen chloride, gallic acid, quercetin, catechin and insulin kit were purchased from Sigma-Aldrich, Saint. Louis, USA. Diagnostic kits used for estimation of lipid profile were procured from Inmesco, Germany. Kits for estimation of urea, creatinine, alanine aminotransferase and aspartate aminotransferase were purchased from Chronolab, Germany. All chemicals and reagents were obtained commercially and were of analytical grade.

**Plant material**

*Guiera senegalensis* roots were obtained from Bagarmiré, Cameroon. The plant specimen was authenticated and deposited at the National Herbarium of Cameroon under the number 41528/NHC. Thereafter, *G. senegalensis* roots were washed with water, chopped, shade-dried at room temperature and powdered.

**Preparation of plant extracts**

**Aqueous extract:**

The fine powder of *G. senegalensis* roots (300 g) was dissolved in 2 l of distilled water and the mixture was boiled for 45 min. The decoction once cooled at room temperature, was filtrated using Whatman No.1 paper and the ﬁltrate was evaporated in an oven at 45°C. The crude extract was weighed (12.25 g) and the extraction yield (4.08% w/w) was calculated.

**Methanol extract**

Powdered roots of *G. senegalensis* (500 g) were macerated using 3 l of 80% methanol for 48 hr under cold conditions. The extract solution was filtrated and concentrated using a rotary evaporator under reduced pressure at 45°C. The process of maceration and evaporation was repeated until exhaustion of the plants. Extracts were combined and dried using an oven (30°C) to completely remove the methanolic solvent. The extraction yield was 3.01% w/w (15.46 g), calculated based on the dry weight of the plant material.

**Phytochemical analysis**

**Qualitative screening**

The preliminary phytochemical screening of *G. senegalensis* extracts was performed to identify the presence of secondary metabolites groups (glycosides, alkaloids, tannins, anthraquinones, saponins, terpenoids, flavonoids, and phenols) using the standard methods (Savithramma et al., 2011 ▶). 

**Quantitative screening**

**Total phenol content**

Folin-Ciocalteu reagent was used for the determination of total phenol content using the method given by Singleton and Rossi (1965). Here, 0.1 ml of dilutions of extracts, 0.5 ml of Folin-Ciocalteu reagent (1/10 v/v dilution) and 5 ml of Na_2_CO_3_ solution (1 M) were mixed together. The mixture was left to stand in the dark at room temperature for 30 min. A standard curve was prepared by using gallic acid (0.1, 0.2, 0.3, 0.4, 0.5 and 0.6 mg/ml) as standard. The experiment was carried in triplicate. The absorbance was read at 727 nm using a UV-visible spectrophotometer (AuCy International Trading Co., Ltd, No. 552 LianCao Road, Minhang District, Shanghai, China). Total phenol content was expressed as milligrams of gallic acid equivalents per mg of dry extract (GAE/gE) using the equation obtained from a calibration curve of gallic acid.

**Total flavonoids content**

Total flavonoids content was evaluated following the method given by Samatha et al. (2012) ▶. In fact, 0.5 ml of dilutions of extracts, 2 ml distilled water and 0.15 ml of NaNO_2_ solution at 5%, were mixed together. The whole was incubated for 6 min and 0.15 ml of AlCl_3_ at 10% solution was added thereto; then, the mixture was left to stand for 6 min. Subsequently, 2 ml of NaOH solution at 4% was added to the mixture. 5 ml of distilled water was added and the whole was thoroughly mixed and allowed to stand again for 15 min. A standard curve was prepared by using quercetin (0.2, 0.4, 0.8, 1, 1.2 and 1.4 mg/ml) as standard. The test was performed in triplicate. The absorbance was measured using a UV-vis spectrophotometer (AuCy International Trading Co., Ltd, No. 552 LianCao Road, Minhang District, Shanghai, China) at the wavelength 420 nm. Flavonoids content was calculated as mg of quercetin equivalent per g of dry extract (mg QE/gE) using the equation obtained from a calibration curve of quercetin. 

**Tannins content**

Estimation of total tannins content was done using the method described by Medini et al. (2018) ▶. In fact, 3 ml of methanol solution at 4%, 1.5 ml of concentrated HCl and 50 μl of extract samples were mixed together. The whole was incubated for 15 min at room temperature. A standard curve was prepared by using cathechin (0.05, 0.1, 0.2, 0.4, 0.8 and 1 mg/ml) as standard. The test was performed in triplicate. The absorbance was measured at 500 nm wavelength using a UV-vis spectrophotometer (AuCy International Trading Co., Ltd, No. 552 LianCao Road, Minhang District, Shanghai, China). The quantity of total tannins was calculated as milligrams of catechin equivalent per g of dry extract (mg CE/gE) using the equation obtained from a calibration curve of cathechin. 

**Animals**

Healthy male albino Wistar rats weighing 250-300 g were used in this study. The animals were procured from the animal house of the Faculty of Sciences, University of Ngaoundere (Cameroon). They were preserved in polypropylene cages under standard laboratory conditions, with respect to temperature (22±2°C), relative humidity (45±5°C) and 12/12 hr day/night cycle. Rats were given standard diet and drinking tap water *ad libitum*. The rats were acclimatized to laboratory conditions for 1 week prior to experimentation. Research on experimental animals was conducted in accordance with the guidelines of the Cameroon National Ethical Committee (Ref No. FW-IRB00001954). 

**Induction of diabetes in rats**

Diabetes was induced by intraperitoneal injection of 110 mg/kg of nicotinamide (dissolved in saline solution) 15 min before the intraperitoneal administration of 65 mg/kg of STZ (dissolved in cold citrate buffer, pH 4.5). After 1 week, rats with a blood glucose level greater than 200 mg/dl were selected for this study (Murugan and Pari, 2006 ▶). 

**Experimental design**

A total of 35 male rats (5 normal rats and 30 diabetic rats) were divided into 7 groups of 5 rats each and treated as follows (Sotoudeh et al., 2019 ▶):

Group 1, Normal control rats received 10 ml/kg b.w. of distilled water. 

Group 2, Diabetic control rats received 10 ml/kg b.w. of distilled water.

Group 3, Diabetic rats received 0.25 mg/kg b.w. of glibenclamide. 

Group 4, Diabetic rats received 200 mg/kg b.w. of the aqueous extract.

Group 5, Diabetic rats received 400 mg/kg b.w. of the aqueous extract.

Group 6, Diabetic rats received 200 mg/kg b.w. of the methanol extract.

Group 7, Diabetic rats received 400 mg/kg b.w. of the methanol extract.

These different treatments were given once daily using an intragastric tube for 4 weeks. Body weight was evaluated at 0, 1, 2, 3 and 4 weeks of treatment period. At the end of the experiment, animals were sacriﬁced under anaesthesia (diazepan 10 mg/kg, i.p. /ketamine 50 mg/kg, i.p.) by cervical decapitation. Blood was collected in anticoagulant tubes and centrifuged at 3000 g at 4°C for 10 min. Plasma obtained was used for the estimation of biochemical parameters. 

After blood sample collection, organs such as the kidneys and liver were removed, rinsed in 0.9% NaCl solution and weighed.

**Measurement of biochemical parameters**

Fasting blood glucose was assessed by an One Touch Ultra Mini glucometer (Life Scan Europe, 6300 zug, Switzerland) using the lateral tail vein of the rats at weeks 0, 1, 2, 3 and 4 of the experiment. Plasma insulin levels were measured using enzyme immunoassay (ELISA) kits. Total cholesterol (TC), triglycerides (TG), low density lipoprotein cholesterol (LDL-c), high density lipoprotein cholesterol (HDL-c), urea, creatinine and the activity of aspartate amino transferase (AST) and alanine amino transferase (ALT) were assayed using commercial kits and an auto-analyzer method.

**Statistical analysis**

The results obtained are represented as mean±standard error of the mean (SEM). The differences between the means were analyzed using one-way ANOVA followed by the Tukey post test (for ALT, AST, lipid parameters, urea, creatinine and organ weights) and two-way ANOVA followed by the Bonferroni post test (for blood glucose and body weight) using Graph Pad Prism software version 5.03. A p<0.05 was considered signiﬁcant. 

## Results

**Qualitative phytochemical screening**

Phytochemical analysis revealed the presence of secondary metabolites such as phenols, flavonoids, tannins, saponins, terpenes and glycosides in the aqueous and methanolic extracts from the roots of *G. senegalensis*. Chemical compounds such as anthraquinones and alkaloids were absents in both extracts.

**Quantitative phytochemical analysis**

Table 1 presents the quantitative phytochemical analysis of *G. senegalensis* extracts. Total phenol content is expressed in terms of gallic acid equivalent (standard curve equation: y=1.757 × + 0.305, R2 = 0.985). Indeed, the aqueous extract displayed the highest quantity of total phenols, compared to the methanol extract.

The concentration of total tannins is expressed in terms of catechin equivalent (CE) (standard curve equation: y=4.050 × + 0.063, R2=0.998). The total tannin content of the aqueous extract was higher as compared to that of the methanol extract. 

Flavonoids content is expressed in terms of quercetin equivalent (standard curve equation: y=0.251 × - 0.022, R2=0.992). The amount of flavonoids was almost similar in both extracts.

**Table 1:** Quantitative phytochemical analysis of *G*.* senegalensis *extracts

**Extract**	**Total phenols ** **(mg ** **GA** **E/gE)**	**Total flavonoids** **(mg QE/gE)**	**Total tannins** **(mg CE/gE)**
Aqueous	34.54±0.23	4.86±0.08	16.81±0.15
Methanol	26.01±0.19	4.47±0.11	7.67±0.22

**Body weight and relative organ weight**

The effect of extracts from the roots of *G. senegalensis* on body weight and the relative organ weights of diabetic animals, is presented in Table 2. The weight of diabetic control animals significantly decreased (p<0.001), compared to the normal control group. However, the administration of different doses of the aqueous extract of *G. senegalensis* and glibenclamide resulted in a significant increase (p<0.001) in their body weight, compared to animals in the diabetic control group. Likewise, a significant (p<0.01) increase in body weight was observed at the dose of 400 mg/kg of the methanol extract. 

Compared to the normal control group, the relative weight of the liver of diabetic control animals, significantly decreased (p<0.001) while the relative kidney weight significantly increased (p<0.001) in rats of the same group. However, the relative weight of the liver of the diabetic animals treated with the glibenclamide and different doses of *G. senegalensis* extracts significantly increased (p<0.001), compared to the diabetic control group. In contrast, diabetic animals treated with glibenclamide and different doses of the methanol extract of *G. senegalensis* showed a significant decrease (p<0.01) in relative weight of the kidneys. This decrease was larger expressed (p<0.001) in the groups of diabetic animals receiving 200 and 400 mg/kg of the methanol extract. 

**Blood glucose and plasma insulin levels**

Compared to the normal control group, a significant increase (p<0.001) in blood glucose and a significant decrease (p<0.001) in plasma insulin levels were noted in the diabetic control group (Table 3). 

However, animals treated with glibenclamide and different doses (200 and 400 mg/kg) of the extracts of *G. senegalensis* showed a significant reduction (p<0.01 to 0.001) in blood sugar during the 4 weeks of treatment, compared to the diabetic control group. Moreover, there was a significant increase (p<0.001) in the insulin level in animals receiving glibenclamide and the aqueous extract of *G. senegalensis*. A significant increase (p<0.05) in the insulin level was noted following administration of the methanol extract at the dose of 400 mg/kg.

**Table 2.** Effect of *G. senegalensis* extracts on body weight and relative organ weights in different groups of rats

** Body weight (g) Relative organ weight (mg/g)**				
**Groups**	**Initial**	** Final**		** Kidneys**
Normal control	268.8±7.3	292.2±4.5	30.83±0.80	5.14±0.24
Diabetic control	278.4±4.5	246.4±1.9***	13.14±0.61***	8.86±0.38***
Diabetic + gliben- clamide (0.25 mg/kg)	261.6±4.5	275.8±4.7^§§§^	20.89±0.63^§§§^	7.02±0.27^§§^
Diabetic + aqueous extract (200 mg/kg)	271.8±6.49	280.6±5.5^§§§^	21.55±0.15^§§§^	5.85±0.25^§§§^
Diabetic + aqueous extract (400 mg/kg)	276.4±5.7	293.0±7.0^§§§^	23.53±0.76^§§§^	6.09±0.24^§§§^
Diabetic + methanol extract (200 mg/kg)	263.8±3.7	248.2±5.9	20.22±0.79^§§§^	7.09±0.24^§§§^
Diabetic + methanol extract (400 mg/kg)	281.8±5.59	270.4±5.1^§§^	16.73±0.91^§§§^	6.95±0.41^§§§^

Each value is expressed as mean±SEM (n=5). Data analysis was performed by ANOVA (one-way or two-way) followed by a *post-hoc* test (Turkey or Bonferroni). ***p<0.001 significantly different when compared to normal control group. §§p<0.01; and §§§p<0.001 significantly different when compared to diabetic control group.

**Biochemical parameters**

Table 4 shows some biochemical parameters evaluated in the plasma of diabetic animals treated with the extracts of roots of *G. senegalensis* for 4 weeks. Indeed, there was a significant increase (p<0.05 to 0.001) in urea, transaminase activity, TC, TG, and LDL-c, and a significant decrease (p<0.001) in HDL-c level in diabetic animals group, compared to the normal control group. 

Compared to diabetic control animals, glibenclamide induced a significant decrease in TC (p<0.01), LDL-c (p<0.001), urea (p<0.01) and the activity of ALT (p<0.01) and AST (p<0.05) and, a significant increase (p<0.05) in HDL-c. A significant decrease in TC (p<0.01; p<0.001), TG (p<0.01; p<0.01), LDL-c (p<0.001; p<0.00), urea (p<0.001; p<0.001) and the activity of ALT (p<0.01; p<0.05) and AST (p<0.001 ; p<0.001), and a significant increase in HDL-c level (p<0.01) were also observed in animals treated with doses of 200 and 400 mg/kg of the aqueous extract of *G. senegalensis, *respectively. 

The methanolic extract (200 and 400 mg/kg) resulted in a significant decrease in TC (p<0.05; p<0.01), LDL-c (p<0.001; p<0.001), urea (p<0.01; p<0.001), and the activity of ALAT (p<0.001; p<0.05) and AST (p<0.05; p<0.0 1), and an increase in HDL-c level (p<0.05). The dose of 400 mg/kg of the methanolic extract caused a significant decrease (p<0.05) in TG level. Only the aqueous extract (400 mg/kg) induced a significant drop (p<0.05) in plasma creatinine level.

## Discussion

T2DM is the most common form of diabetes worldwide (85 to 90%) and is therefore a real public health problem (WHO, 2016 ▶). Injection of the STZ-nicotinamide combination into rodents induces T2DM, which is manifested by the destruction of β cells of pancreas (Hadjzadeh et al., 2017 ▶), a decrease in the use of glucose in tissues and an excessive increase in hepatic glycogenogenesis and glycogenolysis (Devi et al., 2010 ▶). The aim of this work was to explore the blood glucose-lowering activity of *G. senegalensis* in diabetic rats. 

**Table 3:** Effect of *G. senegalensis* extracts on blood glucose and plasma insulin levels in insulin resistant rats

	**Blood glucose level (mg/dl)**						**Plasma insulin (µIU/ml)**
**Groups**	**Before diabetes induction**	**Week 0**	**Week 1**	**Week 2**	**Week 3**	**Week 4**	
							
Normal control	88.2±5.2	89.8±7.3	100.4±4.7	99.2±3.4	84.4±8.3	90.6±5.9	24.8±2.1
Diabetic control	83.4±6.7	274.2±15.2***	283.6±19.3***	244.8±20.6***	318.0±13.8***	262.4±11.7***	11.00±1.3***
Diabetic+glibencla-mide (0.25 mg/kg)	79.0± 3.3	296.0±20.1***	229.2±17.4^§§^	155.8±10.3^§§§^	117.4±6.7^§§§^	111.6±7.6^§§§^	23.2±1.7^§§§^
Diabetic+aqueous extract (200 mg/kg)	89.2±4.9	255.8±7.9***	214.2±12.7^§§§^	145.4±9.9^§§§^	124.0±8.1^§§§^	102.2±5.9^§§§^	28.4±2.0^§§§^
Diabetic+aqueous extract (400 mg/kg)	87.4±3.3	308.2±7.9***	212.4±7.9^§§§^	144.6±11.3^§§§^	117.8±8.5^§§§^	97.6±5.1^§§§^	30.4±2.8^§§§^
Diabetic+methanol extract (200 mg/kg)	92.2± 3.9	283.0±9.7***	220.6±3.1^§§^	170.2±5.5^§§§^	135.4±6.0^§§§^	118.8±10.2^§§§^	19.6± 1,8
Diabetic+methanol extract (400 mg/kg)	98.4±4.7	295.2±7.4***	224.8±11.6^§§^	163.0±17.5^§§§^	134.4±13.1^§§§^	111.0±8.1^§§§^	22.8±2.1^§^

Each value is expressed as mean±S.E.M. (n=5). Data analysis was performed by ANOVA (one-way or two-way) followed by a *post-hoc* test (Turkey or Bonferroni). ***p<0.001 significantly different when compared to normal control group. §p<0.05; §§p<0.01; and §§§p< 0.001 significantly different when compared to diabetic control group.

**Table 4:** Effect of *G. senegalensis* extracts on biochemical parameters in insulin resistant rats at the fourth week

**Groups**	**TC (mg/ml)**	**TG (mg/ml)**	**HDL-c (mg/ml)**	**LDL-c** **(mg/ml)**	**ALT** **(U/L)**	**AST** **(U/L)**	**Urea** **(mg/ml)**	**Cr** **(mg/ml)**
Normal control	87.49±6.86	66.54±2.64	42.38±2.62	31.64±8.41	54.58±5.20	83.16±4.56	31.39±2.80	2.45±0.40
Diabetic control	132.62±2.79***	99.48±5.05**	15.10±1.12***	97.61±20.6***	92.94±4.40***	108.38±3.11*	78.18±5.16***	3.25±0.53
Diabetic+glibencla-mide (0.25 mg/kg)	101.59±4.98^§§^	78.15±2.87	29.41±2.56^§^	56.55±6.47^§§§^	67.43±2.96^§§^	84.38±6.38^§^	53.37±4.85^§§^	2.15±0.13
Diabetic+aqueous extract (200 mg/kg)	97.55±4.35^§§^	69.80±5.07^§§^	31.40±3.04^§§^	52.19±5.93^§§§^	48.95±4.56^§§§^	79.51±4.58^§^	45.61±4.1^§§§^	1.99±0.16
Diabetic+aqueous extract (400 mg/kg)	81.71±6.18^§§§^	66.43±6.61^§§^	32.54±3.14^§§^	35.88±2.91^§§§^	43.53±2.73^§§§^	75.39±5.23^§§^	37.71±5.82^§§§^	1.67±0.19^§§^
Diabetic+methanol extract (200 mg/kg)	106.40±5.25^§^	82.99±8.57	28.56±3.06^§^	61.24±6.48^§§§^	60.82±4.54§§§	82.22±7.20^§^	48.76±3.72^§§§^	2.54±0.14
Diabetic+methanol extract (400 mg/kg)	98.22±6.79^§§^	70.50±3.98^§^	29.26±1.05^§^	54.87±6.29^§§§^	57.71±5.30^§§§^	78.99±5.07^§§^	39.99±3.85^§§§^	2.14±0.19

Each value is expressed as mean±S.E.M. (n=5). Data analysis was performed by one-way ANOVA followed by Turkey’s *post-hoc* test. *p<0.05; **p<0.01; and ***p<0.001 significantly different when compared to normal control group. §p<0.05; §§p<0.01; and §§§p<0.001 significantly different when compared to diabetic control group. TC, total cholesterol; TG, triglycerides; HDL-c, high density lipoprotein cholesterol; LDL-c, low density lipoprotein cholesterol; AST, aspartate amino transferase; ALT, alanine amino transferase; and Cr, creatinine.

The ability of the plant to reduce blood sugar and lipid concentration in diabetics is related to its composition of glycosides, flavonoids, phenols, terpenoids, tannins, coumarins, quinones, saponins and steroids (Ghosh and Konishi, 2007 ▶; Reher et al., 1999 ▶). Tannins and saponins possess potent S-GLUT-1 mediated inhibition of glucose transport (Tiwari and Rao, 2002 ▶). Glycosides have the ability to prevent glycogenolysis when there is excess glucose in the body (Kandan and Govindan, 2017 ▶). Saponins have antidiabetic and hypocholesterolemic effects (Rupasinghe et al., 2003 ▶). Flavonoids act as insulinosecrétagogues and/or insulinomimetics, and have the properties of stimulating the storage of glucose in the liver and muscle tissues (Gupta et al., 2011 ▶). Phenols and flavonoids have inhibitory effects on the activity of digestive enzymes (α-amylase and α-glucosidase) (Ganeshpurkar et al., 2013 ▶). Luo et al. (1999) ▶ showed that terpenoids cause a drop in blood sugar in laboratory animals. In this study, the aqueous and methanol extracts of *G. senegalensis* showed considerable amounts of tannins, phenols and flavonoids. These phytocomponents may in part, be responsible for the strong antidiabetic activity observed. 

T2DM induced by streptozotocin /nicotinamide is often accompanied by a severe decrease in body mass, indicating muscle protein degradation caused by carbohydrate metabolism (Hossain et al., 2011 ▶; Sotoudeh et al., 2017 ▶). In the present study, the administration of the aqueous (200 and 400 mg/kg) and methanolic (400 mg/kg) extracts of *G. senegalensis* protected rats from a drastic drop of body weight. This result might be due to the increase in insulin sensitivity, insulin secretion enhancement and glucose homeostasis control. 

In this study, the increase in glycaemia and the decrease in insulinemia observed in diabetic animals may be due to action mechanism of streptozotocin. In fact, streptozotocin has a toxic effect on β cells of the pancreas, it acts by promoting the formation of free radicals that destroy the cytoplasmic membrane of β cells of the pancreas (Bedoya et al., 1996 ▶). In the present work, the glycemia decreased considerably while the plasma insulin concentration increased in diabetic rats treated with *G. senegalensis* extracts for 28 days. *G. senegalensis* extracts and glibenclamide act by stimulating the secretion of insulin by pancreatic β cells and the storage of glucose in peripheral tissues, and inhibiting glucose consumption by muscle tissues. Our results showed that *G. senegalensis* roots contain antioxidant substances (e.g. phenols and flavonoids) that can protect the pancreas against the cytotoxic effect of STZ and regenerate cells β damaged (Ghorbani et al., 2019 ▶).

Diabetes is often accompanied by an alteration in the lipid profile which can be the origin of the occurrence of cardiovascular diseases (Warnholtz et al., 2001 ▶). In this study, untreated diabetic rats showed a significant increase in the level of CT, TG and LDL-c and a decrease in the level of LDL-c. The administration of glibenclamide and *G. senegalensis* extracts for 4 weeks significantly restored the altered lipid profile. These findings confirm that *G. senegalensis* probably acts by inhibiting the enzyme responsible for cholesterol biosynthesis (i.e. 3-hydroxy-3-methylglutaryl coenzyme A reductase), by stimulating cholesterol hydroxylase (the enzyme for cholesterol conversion to bile acids) or by inhibiting intestinal absorption of triglycerides (Mard et al., 2010 ▶; Fox, 2009 ▶). The restoration of the lipid profile is partly due to the action of chemical compounds (saponins and glycosides) present in the aqueous and methanolic extracts of *G. senegalensis* extracts roots (Marrelli et al., 2016 ▶). 

Uric acid, urea, and creatinine are markers of kidney function in diabetic patients. An increase in the concentration of these parameters in the blood of diabetic animals is associated with renal dysfunction (Dabla, 2010 ▶). The administration of glibenclamide and different doses of *G. senegalensis* extracts caused a significant decrease in urea and creatinine levels in the plasma of experimental animals. *G. senegalensis* has nephroprotective properties and therefore, can prevent diabetic subjects from kidney complications. 

Diabetes is associated with increased kidney weights due to an increase in the rate of protein synthesis and/or decrease in the degradation of renal extracellular components (Zafar and Naqvi, 2010 ▶). The accumulation of these extracellular proteins in the kidney cause the thickening of the glomerular basement membrane, thereby impairing kidney function (Zafar and Naqvi, 2010 ▶). The decrease in kidney weight observed in this study might be due to a reversal of renal hypertrophy induced by plant extracts.

Transaminases such as ALT, AST and ALP are markers of hepatic function. An increase in the activity of these enzymes reflects the damage to the liver (Eidi et al., 2006 ▶; Ahangarpour et al., 2017 ▶). In the present study, plant extracts significantly decreased the activity of ALT and AST and increased liver weight, indicating their hepatoprotective effect. Considering the evidence that implicates hyperglycemia in hepatic damage (Vozarova et al., 2002 ▶), this hepatoprotective effect may be due to hypoglycemic effects of these plant extracts as observed in this work. 

In conclusion, the present work indicates that extracts of *G. senegalensis* and especially, the aqueous extract, caused anti-hyperglycemic, anti-hyperlipidemia, and anti-hypoinsulinemia effects and protected the liver and kidneys from hyperglycemia. Our study therefore confirms that *G. senegalensis* extracts possess potential to be used for the treatment of diabetes and its complications.
